# Giant esophageal epiphrenic diverticulum: presentation and treatment

**DOI:** 10.1590/S1679-45082017RC3954

**Published:** 2017

**Authors:** Marcelo Protásio dos Santos, Denise Akerman, Caio Pasquali Dias dos Santos, Paulo Vicente dos Santos, Marcos Claudio Radtke, Fernando Bray Beraldo, José Eduardo Gonçalves

**Affiliations:** 1 Instituto de Assistência Médica ao Servidor Público Estadual, Hospital do Servidor Público Estadual “Francisco Morato de Oliveira”, São Paulo, SP, Brazil.; 2 Universidade Federal de Sergipe, Aracaju, SE, Brazil.

**Keywords:** Diverticulum, esophageal, Diverticulum, Laparoscopy, Esophageal diseases, Fundoplication, Case reports, Divertículo esofágico, Divertículo, Laparoscopia, Doenças do esôfago, Fundoplicatura, Relatos de casos

## Abstract

Epiphrenic diverticulum is a rare disease associated with esophageal motor disorders that is usually asymptomatic and has a well-established surgical indication. We report a case of giant epiphrenic diverticulum in a 59-year-old symptomatic woman who was diagnosed after underwent complementary exams. Because of her symptoms, the surgical treatment was chosen, and esophageal diverticulectomy was performed along with laparoscopic cardiomyotomy and anterior partial fundoplication.

## RESUMO

O divertículo epifrênico é uma patologia rara associada a distúrbios motores esofágicos e, frequentemente, assintomática, tendo indicações cirúrgicas bem estabelecidas. Relatamos um caso de divertículo epifrênico gigante em paciente de 59 anos, sexo feminino, sintomática, diagnosticada por exames complementares. Devido à sintomatologia, optou-se por tratamento cirúrgico, sendo realizada a diverticulectomia esofágica com cardiomiotomia e fundoplicatura parcial anterior laparoscópica.

## INTRODUCTION

Esophageal diverticula are divided into two form, the traction (pharyngoesophageal), and the pulsion diverticulum (epiphrenic). Epiphrenic diverticula are abnormal saccular protrusions in esophagus throughout muscle layer caused by an increase in intraesophageal pressure. This abnormality is linked to esophageal motor disorders.^( 1 )^ Epiphrenic diverticula are rare and considered false diverticula because they affect only the mucous and submucous layers.^( 2 - 4 )^ In the United States prevalence of this disease is approximately 15/100,000.^( 5 )^ This disease treatment depends on intensity of symptoms and risk of potential complications such as bleeding and perforation. Currently, diverticulectomy with cardiomyotomy and laparoscopic fundoplication is the chosen surgical treatment.^( 3 , 6 )^

## CASE REPORT

**Figure 1:**
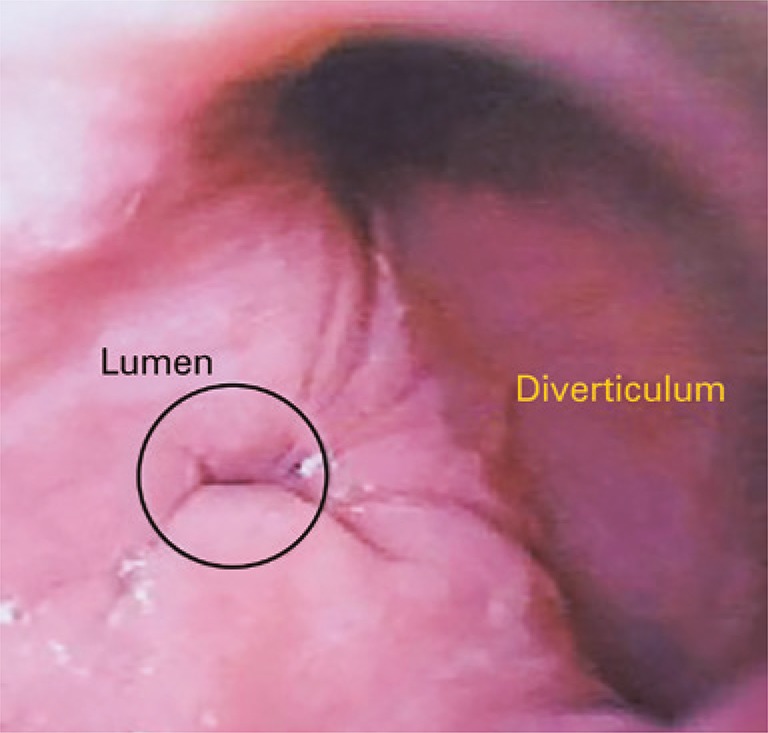
High digestive endoscopy. Epiphrenic diverticulum of large colon near the cardiae

**Figure 2:**
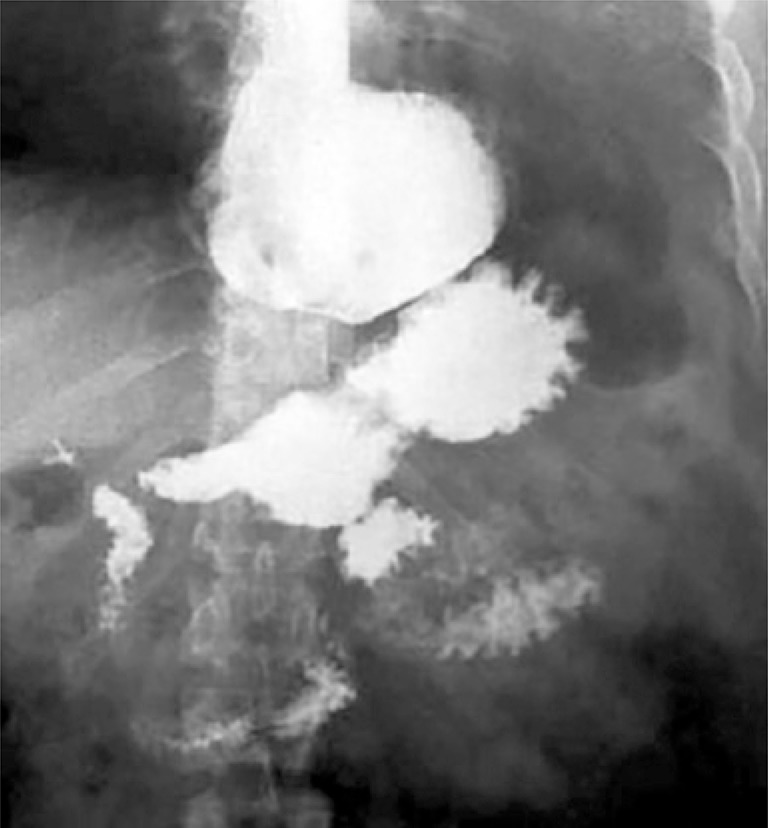
Esophagus, stomach, and duodenum seriography

A 59-year-old woman who complained about epigastric pain, vomiting and postprandial regurgitation, dysphagia and pyrosis for approximately 9 years. The patient reported partial improve of symptoms after treatment with inhibitor of proton bomb. The high digestive endoscopy ( Figure 1 ) showed unique large diverticulum ostium, approximately 2cm in diameter, containing food residues that led to cardiac deviation, and was located in the anterior wall of the distal segment of the esophagus, above the gastroesophageal transition. The esophagus, stomach, and duodenum seriography confirmed the epiphrenic diverticulum ( Figure 2 ). The esophageal manometry revealed hypocontractility of esophageal body (mean amplitude of 20mmHg). Computed tomography of the thorax showed heterogeneity in image content and formation of hydro-air level in the lower posterior mediastinum, anterior to esophagus, greater axis to left in the median line, measuring around 8.1x5.0cm located in greater axial axis. We used laparoscopy for surgery using five punctures technique.

**Figure 3:**
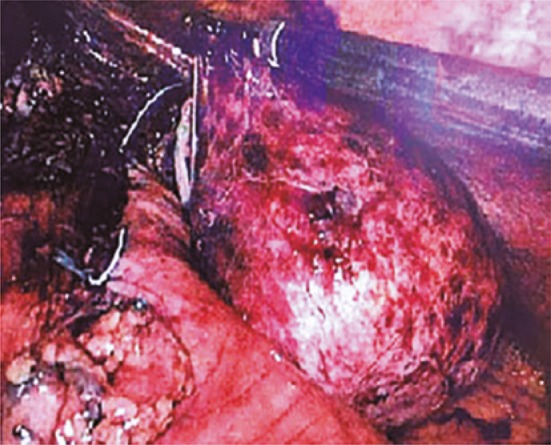
Laparoscopic image of epiphrenic diverticulum

**Figure 4:**
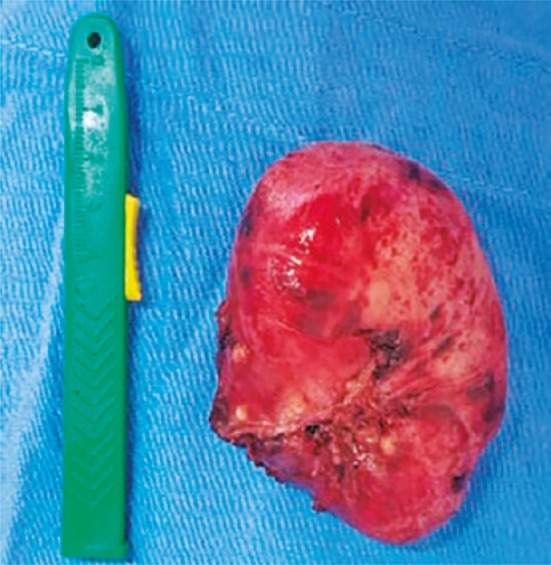
Surgical piece

In the intraoperative period, we found an esophageal diverticulum of approximately 8x7cm, colon of approximately 3cm, and esophageal-gastric transition measuring 8cm ( Figure 3 ). The enlargement of esophageal hiatus was done by opening approximately 1.5cm the diaphragm in order to improve approach of intrathoracic diverticulum. We also did lysis of adhesions of diverticulum with mediastinum using an ultrasonic scalpel. No intercurrences occurred and, subsequently a laparoscopic esophageal diverticulectomy was done for stapling the level of diverticula colon with load of 45mm associated with 5cm cardiomiotomy and laparoscopic anterior partial fundoplicature (pain) to cover the myotomy area and staple line ( Figure 4 ). After surgery, patient reported no symptoms and he had good acceptance of post-surgery diet.

## DISCUSSION

Epiphrenic diverticula are located above the lower esophageal sphincter within 10 to 15cm from distal esophagus; they represent 15% of esophageal diverticula.^( 5 )^ The majority of epiphernic diverticula occur in right posterolateral wall of the esophagus, however, in our case the diverticula occurred in the left anterolateral wall.

Diverticula size ranges from 1 to 14cm, but on average its size is 7.4cm. Symptoms severity are directed related with size.^( 4 )^ Roughly 75 to 80% of patients are asymptomatic.^( 3 )^ When patients are symptomatic, symptoms are dysphagia, regurgitation, nausea and vomiting, pyrosis, halitosis, weight loss, bronchoaspiration, respiratory infections and retrosternal pain.^( 2 , 4 )^

Esophageal motor disorders are closely related with diverticula, mainly achalasia, associated with 3.6 to 7.4% of cases. Epiphrenic diverticulum occurs concomitantly with achalasia in 60% of cases. In such concomitantly cases of esophageal diseases, according to literature, the lack of symptoms in patients without previous history of aspiration and non-dilated esophagus, the need of treatment is excluded. For this reason, patients with small diverticula (smaller than 3cm) treatment can be disregarded, however, greater sizes must be treated.^( 7 )^

Diagnosis can be done by esophagus, stomach, and duodenum seriography, high digestive endoscopy, esophageal manometry and computed tomography.^( 2 , 7 )^

Surgery is indicated when patient is symptomatic or when complications appear, such as bleeding, inflammation, fistulae and mediastinum perforation, or squamous cell carcinoma, which is less common.^( 3 )^

In 1833 Roux performed the first surgery for epiphrenic diverticulum via transabdominal approach, and the first transthoracic resection was done in 1916 by Stierling.^( 3 )^ Because of the association of epiphrenic diverticula with esophageal motor disorders such as achalasia and diffuse esophageal spasms, among other,^( 1 , 8 )^ Effler et al., treated diverticula in the case of myotomy associated with diverticulectomy.^( 3 , 5 , 8 - 10 )^

The Mayo Clinic confirmed this idea with a case series showing that treatment using diverticulectomy only is associated with greater rates of complications and recurrences, comparing with treatment associated with myotomy, being this latter the current indicated treatment.^( 3 , 6 , 7 )^

Mortality after surgery ranges between 0 to 9 and morbidity by around 20%.^( 4 )^ Partial fundoplicature is important to be done to avoid gastroesophageal reflux disease.^( 6 - 8 )^ Main complications of surgical treatment are empyema, abscesses and fistulae that must be rapidly identified and treated. Fails in esophageal myotomy may cause high pressure in staple line of resected diverticulum, and, therefore, breaking the line.^( 3 , 4 )^

Currently laparoscopic is the primary access route because it enables better exposition of gastroesophageal transition, and facilitates myotomy and fundoplicature.^( 6 , 8 )^ Main advantages of laparoscopic route compared with thoracotomy are: higher safety, less postoperative pain, shorter hospitalization and systemic inflammatory response, and fast recovery to daily life activities.^( 5 , 6 )^

## CONCLUSION

Epiphrenic diverticulum is a rare disease, and surgical indication should be carefully evaluated to avoid complications and unnecessary risks. Laparoscopy, if done by experience professionals and specialized service, is the primary procedure for esophageal epiphrenic diverticulum because of its safety and efficiency to solve symptoms. Further medium and long-term studies including large samples are needed to better evaluate results.
